# Extracorporeal shock wave therapy for the treatment of chronic pelvic pain syndrome

**DOI:** 10.1515/med-2020-0174

**Published:** 2020-07-01

**Authors:** Darijus Skaudickas, Titas Telksnys, Vincentas Veikutis, Povilas Aniulis, Mindaugas Jievaltas

**Affiliations:** Medical Academy, Lithuanian University of Health Sciences, Eiveniu 2, 50161, Kaunas, Lithuania; Department of Cardiology, Medical Academy, Lithuanian University of Health Sciences, Kaunas, Lithuania

**Keywords:** chronic pelvic pain syndrome, chronic non-bacterial prostatitis, shock waves, extracorporeal shock wave therapy

## Abstract

**Background:**

Prostatitis is the most commonly diagnosed disease in men younger than 50 years and accounts for about 8% of all urologists’ consultations.

**Objective:**

After evaluating clinical trials and demonstrating the efficacy of chronic non-bacterial prostatitis treatment, it remains of clinical importance to continue studies on the use of low-energy extracorporeal shock wave therapy (ESWT) in men.

**Materials and methods:**

From May 2017 to April 2018, 40 patients with chronic prostatitis (CP) type IIIB/chronic pelvic pain syndrome (CPPS) were enrolled in the study. The patients underwent ESWT once a week for 4 weeks.

**Results:**

The mean age of the patients was 47.8 years. A statistically significant improvement in all the parameters, i.e., the International Prostate Symptom Score (IPSS), the visual analogue scale (VAS), National Institutes of Health Chronic Prostatitis Symptom Index (NIH-CPSI), and the International Index of Erectile Function (IIEF), was observed at week 4. The effect of the treatment was maintained during the entire 12-week period. The NIH-CPSI total score showed the best improvement at week 4, but a slight deterioration without a statistically significant change was noticed at week 12. The greatest improvement at week 4 was documented for the NIH-CPSI and IPSS (43% and 37%, respectively). At week 12, an improvement of 52% and 39% was recorded for VAS and IPSS, respectively.

**Conclusions:**

Our findings confirmed the effectiveness and safety of ESWT in resistant cases of CPPS in the short term. ESWT is cost-effective, which takes little time or requires a small amount of staff, and is easily conducted.

## 1 Introduction

Most men, especially at younger age, experience discomfort in the small pelvic region. Recently, the number of patients suffering from chronic pelvic pain syndrome (CPPS) has been increasing [1]. Patients with chronic non-bacterial prostatitis make up the greatest percentage [2]. Prostatitis is the most commonly diagnosed disease in men younger than 50 years and accounts for about 8% of all urologists’ consultations. According to the published studies, the prevalence of prostatitis in the population ranges from 4% to 11% [3]. Episodic pelvic pain and erectile dysfunction, such as functional CPPS symptoms, mostly affect the quality of life (QoL) of men with this disease [4].

Most probably, the etiology and pathogenesis of chronic prostatitis (CP)/CPPS include a pluricausal, multifactorial mechanism. Perineal and/or pelvic trauma, infection, reflux of some toxic or immunogenic urine material, and/or psychological stress, being an initial stimulus, trigger a cascade of incidents in anatomically or genetically sensitive men, leading to a local reaction of inflammation and/or neurogenic damage. Later on, immunologic and/or neuropathic mechanisms mediated by neuroendocrine pathways propagate or maintain the chronicity of the primary event. All this results in the clinical manifestation of chronic perineal/pelvic pain symptoms related to local and central neuropathic mechanisms [5,6,7,8,9].

**Figure 1:**
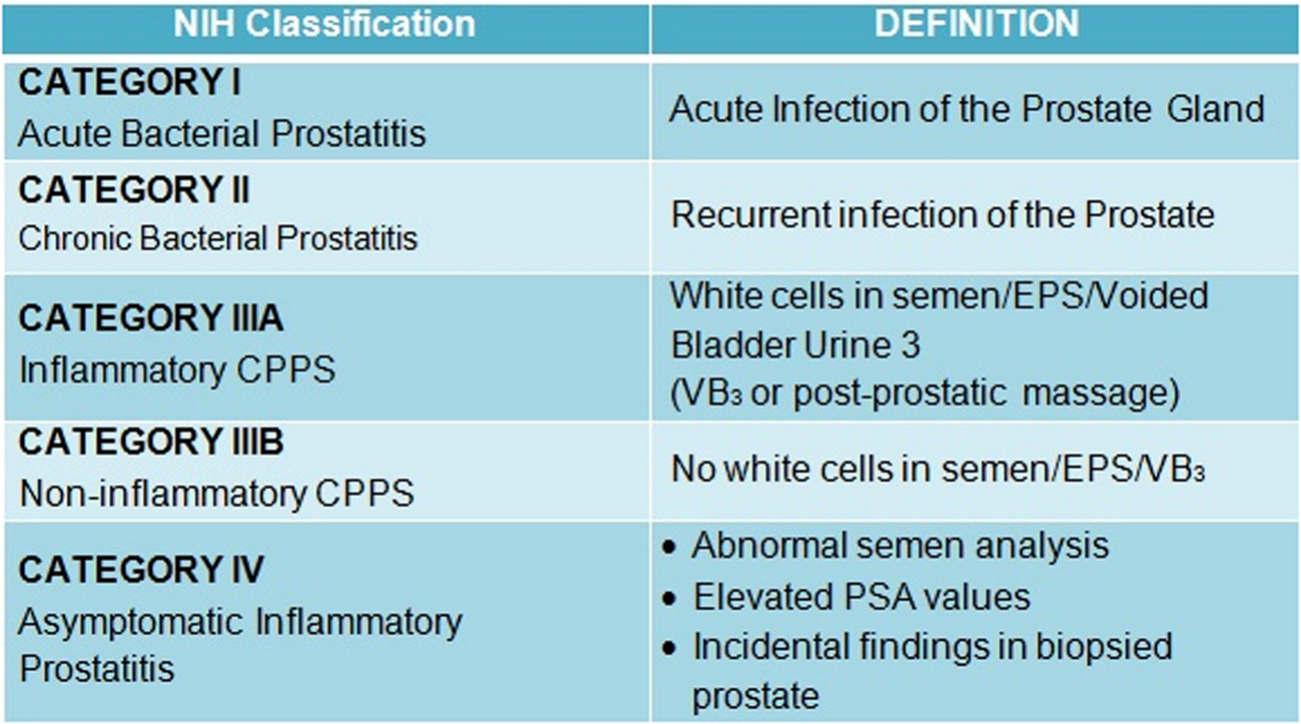
Prostatitis classification of the NIH.

Prostatitis of category III, known as CP/CPPS as well, is the most prevalent type (Figure 1). According to the existing NIH description, CP/CPPS is characterized by genitourinary pain with or without voiding symptoms unaccompanied by uropathogenic bacteria. CP/CPPS is categorized into IIIA and IIIB. Category IIIA refers to the existence of any amount of white blood cells in the semen, post-prostate-massage urine sample, or expressed prostatic secretions. This matches the previously used classification of non-bacterial prostatitis. Category IIIB is similar to the before-used term prostatodynia and is characterized by pelvic pain but no inflammation in the semen [6].

After evaluating clinical trials and demonstrating the efficacy of this treatment, it remains of clinical importance to continue studies on the use of low-energy extracorporeal shock wave therapy (ESWT) in men. A clinical trial of such kind has not yet been performed in Lithuania.

## 2 Materials and methods

From May 2017 to April 2018, 40 patients with CP type IIIB/CPPS diagnosed based on the NIH International Prostatitis Collaboration Network report [10], referred to the Clinic of Urology, Lithuanian University of Health Sciences, were included in this study. All the patients provided signed informed consent. The Bioethics Centre of the Lithuanian University of Health Sciences granted approval to conduct this study (No. BEC-MF-334).

**Figure 2:**
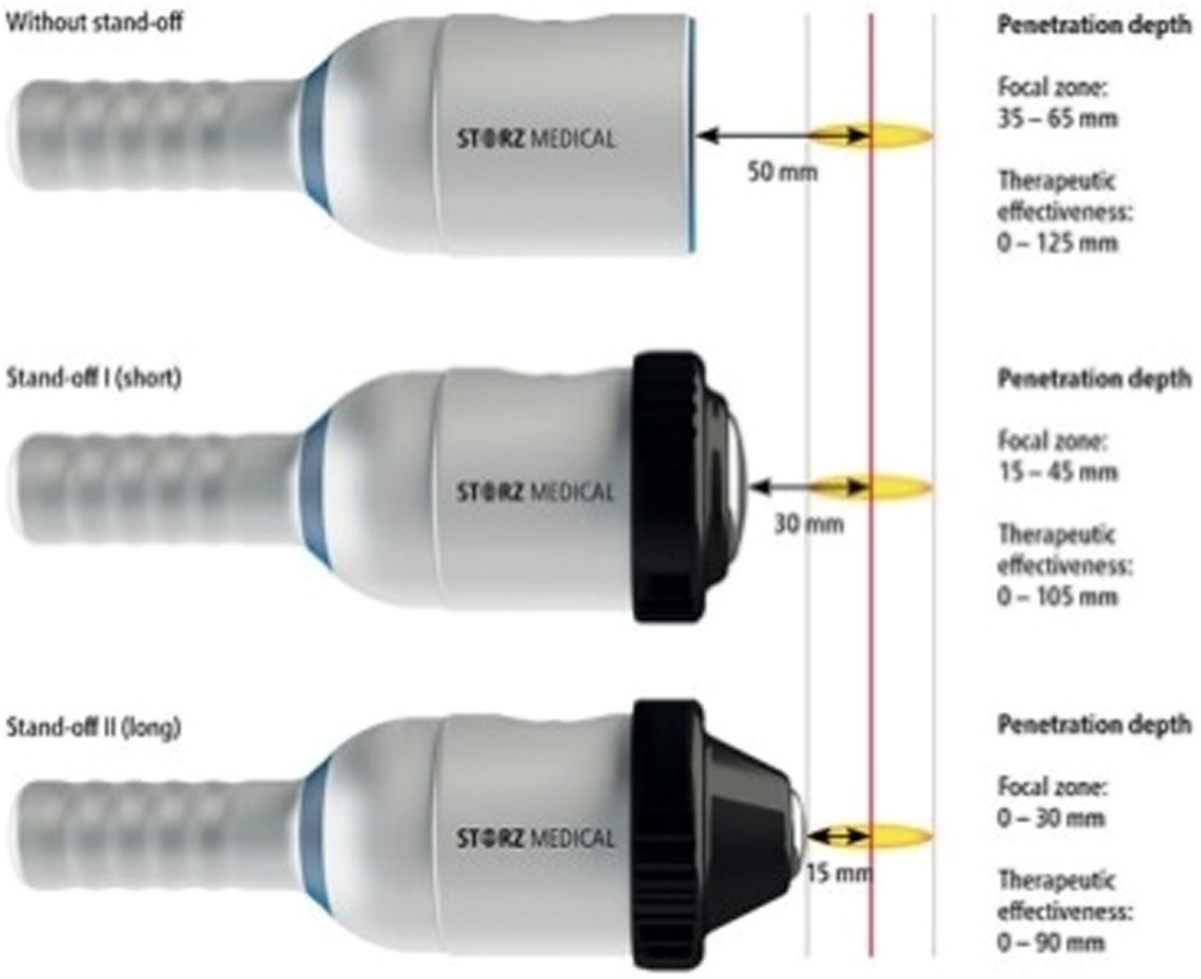
Characteristics of the transducer.

Patients underwent ESWT once a week for 4 weeks (3,000 individually with a maximum total energy flow density of 0.25 mJ/mm^2^, rate 3 Hz). The appliance used to treat patients in this study was a typical electromagnetic shock wave device with a focused shock wave source (Duolith model SD1, Storz Medical, Switzerland). The depth of the transducer focus zone penetration ranges from 35 to 65 mm (Figure 2). The location of the transducer was changed every 500 pulses perineally to affect the whole prostatic and pelvic floor region.

The effect of ESWT on pain, QoL, erectile function, and urination was estimated. These parameters were evaluated using the following validated questionnaires: the National Institutes of Health Chronic Prostatitis Symptom Index (NIH-CPSI), the International Prostate Symptom Score (IPSS), the International Index of Erectile Function (IIEF), and the visual analogue scale (VAS).

Data were compared using the paired-samples *t* test. The statistical package IBM SPSS Statistics, version 21, was used for statistical analysis.

## 3 Results

The mean age of the patients was 47.8 ± 11.1 years (range 25–78). The patients were not categorized by the type of treatment they received prior to ESWT because it was very different and individual. Table 1 depicts the results of our study after treatment.

**Table 1:** Results: changes in parameters

Parameter	*P* value	95% CI	Mean difference	Improvement, %
IPSS (week 0) – IPSS (week 4)	<0.001	2.3 to 3.4	2.8	37
IPSS (week 0) – IPSS (week 12)	<0.001	2.2 to 3.9	3.0	39
IPSS (week 4) – IPSS (week 12)	0.367	−0.3 to 0.7	0.2	4
VAS (week 0) – VAS (week 4)	<0.001	0.4 to 0.7	0.5	24
VAS (week 0) – VAS (week 12)	<0.001	0.9 to 1.4	1.1	52
VAS (week 4) – VAS (week 12)	<0.001	0.4 to 0.8	0.6	38
NIH-CPSI (week 0) – NIH-CPSI (week 4)	<0.001	3.7 to 6.5	5.1	43
NIH-CPSI (week 0) – NIH-CPSI (week 12)	<0.001	2.9 to 6.2	4.5	38
NIH-CPSI (week 4) – NIH-CPSI (week 12)	0.225	−1.5 to 0.4	−0.6	−9
IIEF (week 0) – IIEF (week 4)	<0.001	−1.6 to −0.5	−1.1	6
IIEF (week 0) – IIEF (week 12)	<0.001	−1.9 to −0.9	−1.4	8
IIEF (week 4) – IIEF (week 12)	0.230	−0.9 to 0.2	−0.3	2

IPSS, International Prostate Symptom Score; VAS, visual analogue scale; NIH-CPSI, National Institutes of Health Chronic Prostatitis Symptom Index; IIEF, the International Index of Erectile Function.

A statistically significant improvement in all the parameters, i.e., IPSS, VAS, NIH-CPSI, and IIEF, was observed at week 4. The effect of the treatment remained during the entire 12-week period. Meanwhile, the NIH-CPSI total score showed the greatest improvement at week 4, but a slight deterioration without a statistically significant change was noticed at week 12.

**Figure 3:**
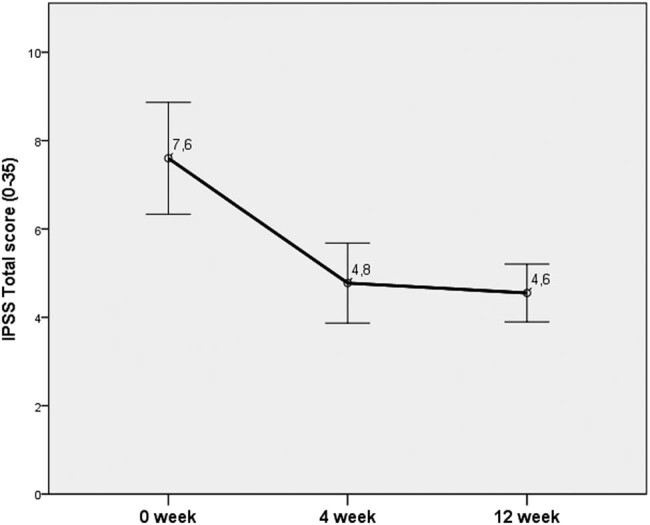
Changes in the total score of IPSS.

**Figure 4:**
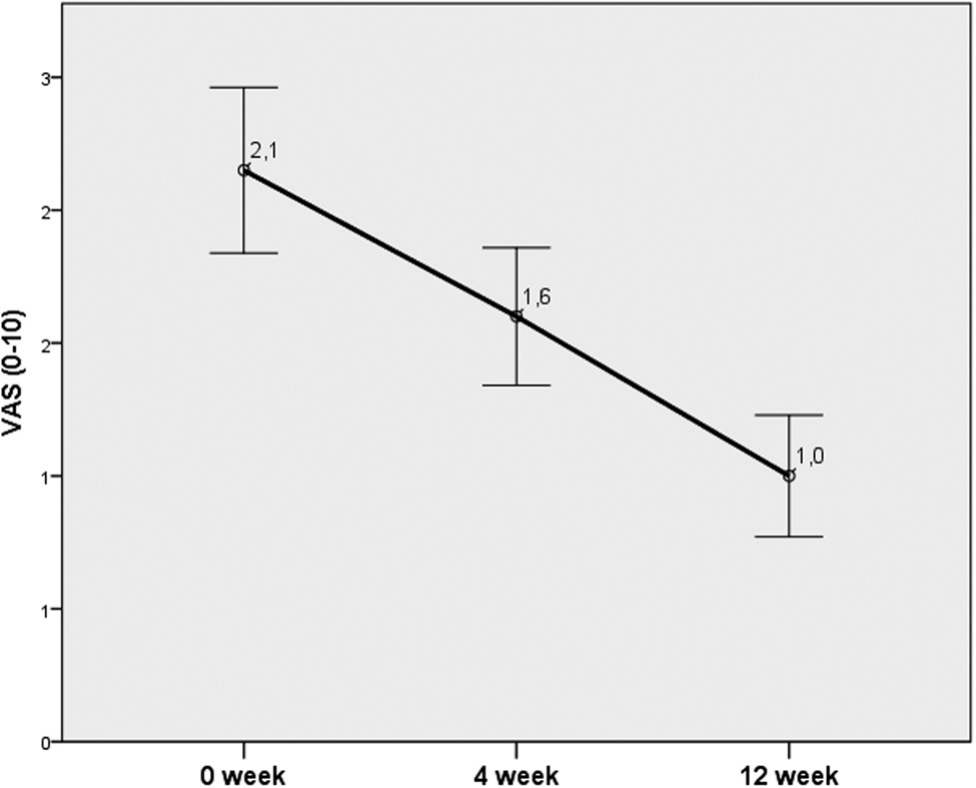
Changes in the VAS score for pain.

**Figure 5:**
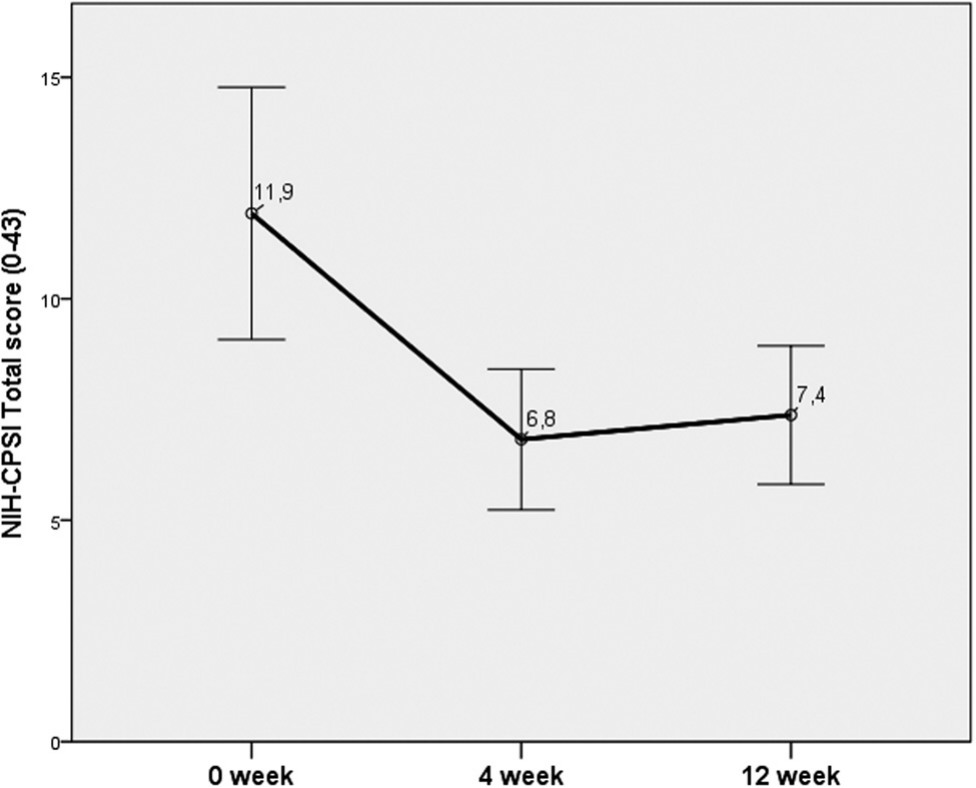
Changes in the NIH-CPSI.

**Figure 6:**
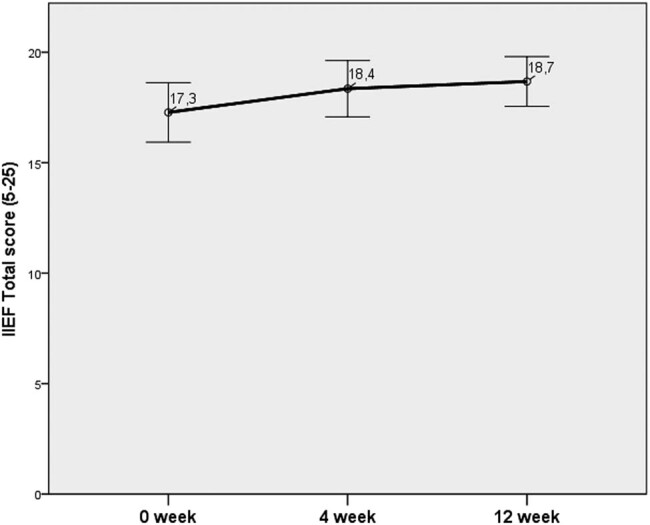
Changes in the IIEF.

At week 4, the greatest improvement was documented for the NIH-CPSI and IPSS (43% and 37%, respectively). At week 12, an improvement of 52% and 39% was recorded for VAS and IPSS, respectively (Figures 3–6).

No side effects occurred in any patient through the treatment and follow-up periods.

## 4 Discussion

The treatment of CPPS is multidisciplinary. There are no precise treatment recommendations on how to cure this problem, which has a significant impact on the QoL not only of men but also of their partners. Urinary disorders, pain syndrome, and erectile dysfunction indirectly affect close relatives of patients. Antibiotics, analgesics, α-blockers, antiphlogistics, 5-alpha-reductase inhibitors, and homeopathic agents are used for individual combinations [11]. Physiotherapy, acupuncture, trigger-point massage, electromagnetic treatment, traditional Chinese medicine, rectal massage, thermotherapy, hyperthermia, invasive neuromodulation, balloon dilatation, laser coagulation, and intraprostatic injection of botulinum toxin A could be mentioned as alternative strategies for the treatment of CPPS [12,13].

Currently, low-energy ESWT in clinical settings is used to heal orthopedic pain syndromes, fractures, and wounds. Several mechanisms on how low-energy ESWT could affect CPPS have been proposed, namely reduction in passive muscle tone, hyperstimulation of nociceptors, blockage of the flow of nerve impulses, or the effect on the neuroplasticity of pain memory [14]. Other studies have shown that shock waves are simply applicable perineally without side effects, with a significant improvement in CPPS symptoms, especially in patients with expanding and repeating pain [15,16].

According to our literature review, Zimmermann et al. in their study [14] conducted in 2008 reported that pain and QoL considerably improved after ESWT. An improvement in voiding conditions, but not statistically significant, was also recorded. They found that only 17% of the patients showed an increase in serum the prostate specific antigen (PSA) 2 days after the treatment, and this shows that ESWT is not traumatic for the prostate gland. In our study, no pain or discomfort was documented during or after the treatment as well.

Zimmermann et al. in their later study [15] showed that all patients who underwent ESWT reported significantly reduced pain, better QoL, and improved voiding conditions as compared to the placebo group.

A study by Moayednia et al. [16] showed ESWT to be a safe and efficient procedure in patients suffering from CPPS during short-term follow-up, but no long-term efficacy of ESWT was documented. Contrary to the study by Moayednia et al. [16], Al Edwan et al. [17] reported that the long-term efficacy of ESWT remained within 12 months despite a statistically insignificant deterioration observed after 2 weeks.

In a randomized study by Yan et al. [18], involving 80 patients with CPPS, a significant improvement in the NIH-CPSI, QoL, and pain was observed in comparison with the baseline at all post-treatment results in the ESWT group.

A study by Guu et al. [19] reported extremely good results using ESWT in patients who were unsuccessfully treated with a traditional therapy previously. A total of 33 patients who received at least a 6-week trial of triple antibiotic therapy, including fluoroquinolone, alpha blocker, and acetaminophen (a non-steroidal anti-inflammatory drug), were included in the study. These patients achieved a clinically significant improvement in a short time after the ESWT therapy. The advantage of this study is that the selection criteria were homogenous. In our study, the patients were not categorized by the type of treatment they received prior to ESWT because it was very different and individual.

Pajovic et al. [20] conducted a study aiming at determining the effect of combination of ESWT and triple therapy versus triple therapy alone in patients with category IIIB CP/CPPS and showed a cumulative effect of adding ESWT to the standard treatment. Patients who were administered triple therapy showed no significant improvement in post-void residual urine and maximum flow rate (*Q*_MAX_); meanwhile, the combination treatment resulted in both significantly improved post-void residual urine and *Q*_MAX_ values. All items of the NIH-CPSI improved statistically significantly in both groups after the treatment, but better results were documented in the group that was administered the combination of ESWT and triple therapy.

In our study, a significant improvement in the IPSS, VAS, NIH-CPSI, and IIEF was documented at week 4. The efficiency of the treatment was retained during the whole period of 12 weeks. Pain that is considered the main parameter deteriorating the QoL was significantly reduced in this study.

## 5 Conclusions

In conclusion, our findings confirmed the effectiveness and safety of ESWT in resistant cases of CPPS in the short term. ESWT is cost-effective, which takes little time or requires a small amount of staff, is easy to conduct, and prevents systemic adverse effects. ESWT is a local therapy with the opportunity of repeating the treatment at any time. Furthermore, large-scale and long-term studies are required to compare the efficacy of and to describe a standard protocol for ESWT.

The limitations of the design of our study are that there is no randomized controlled group and that our study is a non-blinded single-arm clinical trial. We are thinking that ESWT may have psychologically affected the clinical outcome.

Furthermore, large-scale and long-term studies with a control group are required to compare the efficacy of and to describe a standard protocol for ESWT.
